# Environmental Assessment of Friable Asbestos from Soil to Air Using the Releasable Asbestos Sampler (RAS)

**DOI:** 10.3390/toxics10120748

**Published:** 2022-12-01

**Authors:** Puteri Tiara Maulida, Jeong Wook Kim, Myung Chae Jung

**Affiliations:** Environmental Geochemistry Laboratory, Department of Energy Resources and Geosystem Engineering, Sejong University, 209, Neungdong-ro, Gwangjin-gu, Seoul 05006, Republic of Korea

**Keywords:** releasable asbestos sampler (RAS), friable asbestos, abandoned mining area, environmental assessment, excess lifetime cancer risks

## Abstract

The objectives of this study are to examine the feasibility of the releasable asbestos sampler (RAS) equipment for laboratory tests as an alternative to activity-based sampling (ABS), and to apply the equipment controlled by wind velocity and water contents in the field to asbestos-contaminated soils. Two asbestos-contaminated mines (the Jecheon mine and the Jongmin-ri mine) were selected. At each mine, 21 surface soils (0~15 cm) were sampled, the asbestos concentrations were analyzed, and then three representative sites, containing 0.25%, 0.50%, and 0.75% of asbestos in soils, were chosen to evaluate the amount of releasable asbestos by the modified RAS with wind velocity and water contents. The results showed that the levels of releasable asbestos from soil to air increased with higher wind velocities and lower water content. In addition, the application of risk assessment of releasable asbestos in the soils as an alternative to the activity-based sampling (ABS) method was established at each site, and an estimation of the excess lifetime cancer risk (ELCR) was also calculated. According to the calculation, the estimated ELCR values did not exceed the threshold value (1 × 10^−4^) in the Jecheon mine for all the soils, while some samples from the Jongmin-ri mine exceeded the threshold value. Therefore, proper remediation work is needed to control friable asbestos from soils to air in the vicinity of the mines.

## 1. Introduction

Asbestos is a naturally occurring fibrous silicate mineral and has been used in a variety of insulation, fireproofing, and building materials due to its fiber strength and heat resistance. It can be categorized into two groups, both serpentines and amphiboles. Chrysotile classified as the serpentine group with magnesium silicate is the most common type of asbestos, whilst the other five remaining minerals, including crocidolite (fibrous riebeckite), amosite (fibrous grunerite), anthophyllite, tremolite, and actinolite, are all amphibole groups with ferromagnesium silicates [1,2,3]. Despite its global use, asbestos derived from both naturally occurring asbestos (NOA) and anthropogenic sources have been forbidden from use because it effects harmful health problems, especially lung cancer, and thus the use of asbestos was banned and/or restricted in 55 countries worldwide [4,5,6,7]. For example, the US Environmental Protection Agency (US EPA) was concerned and started moving towards an asbestos ban in 1979, and the issue was transferred to the Federal Occupational Safety and Health Agency (OSHA) and the Consumer Product Safety Commission (CPSC) in 1984 [8]. Eventually, the use of asbestos was banned by the US EPA in 1989. However, it is still present in various structures built before that the time.

Because asbestos exposure is not a problem if solid asbestos is left alone and not disturbed, an activity-based sampling (ABS) method was used as the preferred approach for assessing asbestos exposure at asbestos-contaminated sites [9]. The sampling methods, however, recognized that static or fixed area sampling would not adequately capture personal exposure. Thus, the releasable asbestos field sampler (RAFS) was developed as an alternative to ABS, which is personal breathing zone sampling during a stimulated activity [10]. The RAFS was designed to analyze an accurate emission rate and concentration from soils operating under actual soil conditions with wind velocity, water content, and soil texture. In addition, the RAFS is easy to collect asbestos releasability data under both laboratory and field conditions, and performs well in all weather conditions [10].

There are various types of research related to the environmental risk assessment of asbestos in rocks and soils, including analytical techniques [11,12,13], the activity-based sampling (ABS) method [14], animal and human health [15,16], and effects on mining activity [17,18]. In addition, several countries have set standard regulations on operating procedures of asbestos, including the US EPA [8,19], Australia [20], the Netherlands [21], South Korea [22,23], and other countries. For example, South Korea has set regulations for the investigation and remediation methods of asbestos in rock and soil [24]. As a regulation, remediation work of abandoned asbestos mines in South Korea has been implemented a few years before due to the harmful effects of its pollution on human health due to breathing in the surrounding area if the asbestos fibers are released into the air as an asbestos exposure [23,24]. Thus, to estimate the risk assessment of asbestos, an excess lifetime cancer risk (ELCR) was applied to the scenarios using both the US EPA Integrated Risk Information System (IRIS) and the California EPA Office of Environmental Health Hazard Assessment (OEHHA) [25,26].

Therefore, the objectives of this study were to examine the validity of the modified RAS equipment by a laboratory test and to apply it in the field controlled by wind velocity and water content in asbestos-contaminated soils. This study also examined the application of risk assessment of asbestos by estimating ELCR at the two represented abandoned mine sites using operating data by the modified RAS. The results of this study can be used as basic data for environmental impacts and damage compensation of residents for asbestos contamination in the future. In addition, it can be used as supporting data for the quick evaluation and appropriate evaluation of the degree of exposure to friable asbestos contained in the soils.

## 2. Materials and Methods

### 2.1. Study Areas and Soil Sampling

In South Korea, there are approximately over 5500 mines, both 2500 metalliferous mines and 3000 non-metal mines, and most of the mines were abandoned due to economic recession. Among the mines, only 38 mines are counted as asbestos mines and all the mines are closed now. After closing the mines, a large number of mine wastes containing asbestos were left without proper remediation work. Two abandoned mines were selected, both the Jecheon mine and the Jongmin-ri mine, which are included in national priority remediation sites decided by a national survey of asbestos contamination in soils undertaken by the Korean Ministry of Environment (KME) [24].

**Figure 1:**
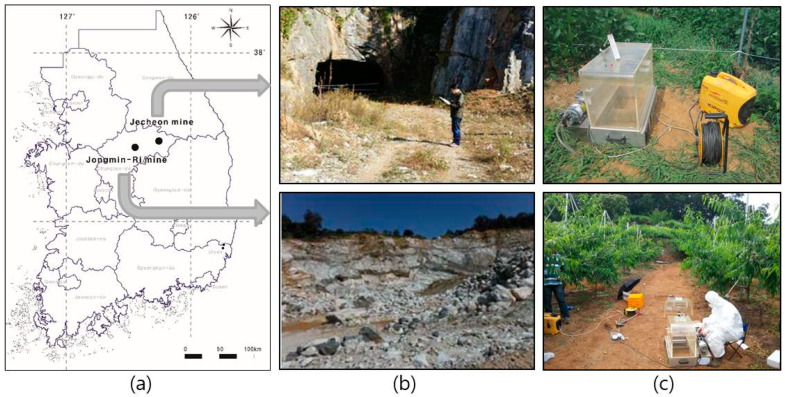
Locations (**a**), mining sites (**b**), and experiment of the modified releasable asbestos sampler (RAS) in the field (**c**).

The locations of the two study areas are shown in Figure 1. As shown in the figure, the Jecheon mine is located in Jecheon city, Chungbuk, South Korea (latitude: 36°55′38.32″, longitude: 128°09′51.34″). During the mine operation in the 1970s, the mine produced various minerals including asbestos, fluorite, and limestone. Recently, the mine was prepared for remediation work by the Korean government.

The Jongmin-ri mine is located in Chungju city, Chungbuk, South Korea (latitude: 37°00′15.09″, longitude: 127°59′01.99″). The mine originated from Cu mineralization found in 1965, and then talc mineralization associated with asbestos was exploited in the 1980s. After finishing talc production in 2000, the mine also produced a total of 36,000 tons of limestone from 2009 to 2012. At present, the mine exploration stopped and a large amount of asbestos-contained waste was left without proper remediation work.

At each mine, 21 surface soils (0~15 cm) were sampled within the distance of 1.5 km from the Jecheon mine and 1.0 km from the Jongmin-ri mine. Asbestos concentration, water content, and soil texture of the samples from the mines are summarized in Table 1. As shown in the table, the average concentrations of asbestos and water contents in the soil from the Jecheon and Jongmin-ri mines are similar to 0.42 and 0.43%, and 18.9 and 21.4%, respectively. As a result of soil texture analysis, the soils are classified as loam and silt loam at the Jecheon mine and sandy loam at the Jongmin-ri mine.

Among the soil samples, three representative soils containing 0.25%, 0.50%, and 0.75% of asbestos were selected to examine the releasability of asbestos by the modified RAS from the RAFS developed by the US EPA [10]. Detailed information on the selected soil samples is also shown in Table 1.

### 2.2. Instrument and Analysis

#### 2.2.1. The Modified RAS Equipment

The releasable asbestos sampler (RAS) equipment used in this study was modified from a self-made version of the RAFS instrument developed by the US EPA [10], which is designed as an alternative to the activity-based sampling (ABS) method for risk assessment of asbestos-contaminated soils. This study attempted to develop a modified RAS that replaces the weeding and digging of the ABS, which are the most important activities in the agricultural area of Korea. In addition, the two scenarios (weeding and digging) can be controlled by wind velocity and water contents. Thus, this study examined the friable asbestos concentrations from soil to air by the modified RAS with wind velocity and water contents.

**Figure 2:**
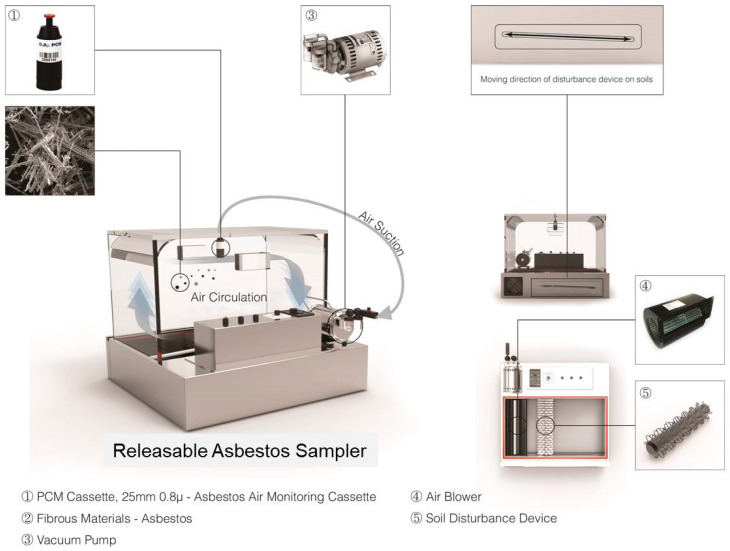
The modified releasable asbestos sampler (RAS) equipment adopted in this study. The sampler consists of three parts: (1) a soil disturbance device (⑤) with a horizontal-typed air blower (④), (2) a box made by the acrylic panel in a dimension of 30 cm (W) × 50 cm (L) × 50 cm (H), and (3) an asbestos sample collector by PCM cassette (①) with a vacuum pump (③).

As shown in Figure 2, the modified RAS consists of three parts: (1) a soil disturbance device (⑤) with a horizontal-typed air blower (④), (2) a box made by the acrylic panel in a dimension of 30 cm (W) × 50 cm (L) × 50 cm (H), and (3) an asbestos sample collector by PCM cassette (①) with a vacuum pump (③). The asbestos attached to the soil was separated by the wind blowing from the air blower attached to the bottom of the modified RAS equipment and then circulated in the acrylic panel. The released asbestos was collected through a sample collector using a 25 mm mixed cellulose ester membrane filter connected to a vacuum pump.

Both laboratory tests and field tests were carried out to validate the modified RAS in this study several times. The test included checking the air-blowing velocity controlled by a horizontal-typed fan, the degree and extent of circulation of asbestos as fine particles in the containing box controlled by wind velocity (0 to 10 m/s) and water contents (0~30%), the best position of the sample collector (upper, lower, left, center, and right, etc.) in the box, and the effectiveness of the sample collector as air pump pressure for adjusting the volume of air samples.

#### 2.2.2. Analysis of Asbestos in Soils and Friable Asbestos from Soil to Air

Two representative mines, the Jecheon mine and the Jongmin-ri mine, contaminated by asbestos due to mining activities, were selected to examine the possibility of the modified RAS as an alternative to the ABS and to evaluate the risk assessment of releasable asbestos from soil to air. At each mine, three representative sites containing 0.25%, 0.50%, and 0.75% of asbestos in surface soils (0–15 cm) were sampled, with an amount of over 5 kg placed into the clean polyethylene bag (wrapped). All samples were transported to the laboratory and then dried at 40 °C for 3 days using an air-controlled dry oven. The dried samples were placed into a slow-moving agitator for homogenization sampling for 7 days. After homogenizing, the samples were wrapped into the clean polyethylene bag and stored in cool condition.

Soil moisture content is critical when utilizing the modified RAS that tests the releasability of asbestos from the soil. Thus, soil moisture contents were directly measured by the soil moisture meter (DM-18, Takemura Electric Works LTD, Tokyo, Japan) at each experiment. To establish the validation, moisture contents were adjusted by adding deionized water into the samples (500 g of dry weight) with spray until gaining moisture of 10~20% and 20~30% in the acrylic panel. In general, asbestos in soils with over 35% of water content is not able to be released. Wind velocity could be controlled by the flow rate during the collection of samples into the cassette. Various wind velocities (0, 1, 2, 3, and 5 m/sec) were adjusted by the horizontal-typed fan with an air blower.

Asbestos in soil samples was measured by polarizing lighting microscope (PLM) using the 400-points counting method. The method is currently a standard method applied to soil asbestos analysis in South Korea, defined as 0.25% (=1/400) when one asbestos fiber is observed out of the 400-points measurements, and 0.50% (=2/400) if two asbestos fibers are observed in PLM. In addition, asbestos concentrations in the air collected by the modified RAS were analyzed by a phase-contrast microscope (PCM), according to the National Institute for Occupational Safety and Health (NIOSH) Method 7400A and ISO 10312:1995 (E) counting rules [24].

#### 2.2.3. Calculation of ELCR

The US EPA Superfund program defines the acceptable risk range for exposure to a carcinogen, such as asbestos, as 10^−4^ excess lifetime cancer risk (ELCR). Thus, an estimated exposure value of more than 1 in 10,000 excess cancers is considered to be one of concern and may require proper action to reduce the exposure and resulting risk in ELCR measurement. The ELCR is the risk assessment that presents quantitative estimates of excess cancer risk over a lifetime in a population based on the defined exposure scenarios [25]. For the calculation, the general equation to estimate risks from inhalation of asbestos is:

ELCR = EPC × TWF × IUR(1)

where ELCR is the excess lifetime cancer risk, the risk of developing cancer as a consequence of the site-related exposure; EPC is the exposure point concentration, the concentration of asbestos fibers in the air (f/cc) for the specific activity being assessed; IUR is the inhalation unit risk (f/cc)^−1^; and TWF is the time weighting factor, which accounts for less-than-continuous exposure during a one-year exposure and is obtained by:

(2) $$\mathrm{TWF}=\frac{\mathrm{Exposure}\;\mathrm{time}\;\left(\mathrm{hours}/\mathrm{day}\right)}{24}\times \frac{\mathrm{Exposure}\;\mathrm{frequency}\;\left(\mathrm{days}/\mathrm{year}\right)}{365}$$

## 3. Results and Discussion

### 3.1. Friable Asbestos Levels with Wind Velocity and Water Contents

The type of asbestos in soils from the two study mines is uniformly found to be fibrous tremolite (Ca_2_(Mg)_5_(OH)_2_Si_8_O_22_), non-colored or yellow, with the refractive index of 1.60~1.64, the birefringence of 0.02~0.03, the aspect ratio of over 3:1, and the average length of 7.2 μm with a range of 5.0 to 10.3 μm. Tremolite is a member of the amphibole group of silicate minerals, and it is formed mainly by the metamorphism of sediments rich in dolomite and quartz. It is known to be toxic, and inhaling the fibers can lead to asbestosis, lung cancer, and both pleural and peritoneal mesothelioma. Fibrous tremolite is sometimes found as a contaminant in vermiculite, chrysotile, and talc [26,27,28].

Through this treatment, it will be possible to know whether more or less releasable asbestos with higher moisture content can be obtained. While the modified RAS equipment runs, friable asbestos would be released into the air from the soil surface and goes through the cassette with 25 mm mixed cellulose ester (MCE) membrane filters and an air pump speed of 15 L/min for 27 min running time (total volume of 400 L).

**Figure 3:**
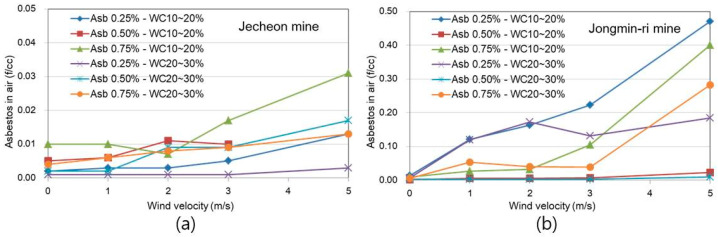
Asbestos contents in the air with asbestos contents in soils (Abs with 0.25%, 0.50%, and 0.75%), wind velocity (0, 1, 2, 3, and 5 m/s), and water contents in soils (WC of 10~20% and 20~30%) controlled by the modified RAS equipment adopted in the Jecheon mine (**a**) and the Jongmin-ri mine (**b**) areas.

Asbestos contents in the air, released by soil asbestos contents of 0.25%, 0.50%, and 0.75%, wind velocity of 0, 1, 2, 3, and 5 m/s, and soil water contents of 10~20% and 20~30%, controlled by the modified RAS equipment adopted in the study area are shown in Figure 3, and additional data can be found in the Supplementary Materials of Table S1. As shown in the figure for the Jecheon mine, the maximum level of released asbestos from soils was 0.013 f/cc under the conditions of 10~20% of water content and 5 m/s of wind velocity with 0.25% asbestos-containing soils. In the case of 0.50% asbestos in soil, the maximum level of friable asbestos was 0.018 f/cc at 10~20% water content and 5 m/s wind velocity. In addition to 0.25% asbestos in soil, the maximum level of friable asbestos was 0.031 f/cc at 10~20% water content and 5 m/s wind velocity.

In line with the results from the Jecheon mine, the Jongmin-ri mine also showed the levels of releasable asbestos from soil to air increased with higher wind velocities and lower water content. In soils containing 0.25% asbestos from the Jongmin-ri mine, the maximum level of friable asbestos was 0.023 f/cc at 10~20% water content and 5 m/s wind velocity. In the case of 0.50% asbestos in soil, the maximum level of friable asbestos was 0.400 f/cc at 10~20% water content and 5 m/s wind velocity. In addition to 0.75% asbestos in soil, the maximum level of friable asbestos was 0.471 f/cc at 10~20% water content and 5 m/s wind velocity.

In comparison to asbestos concentrations in surface soils from the two mining sites, relatively high asbestos concentrations were found in the soils from the Jongmin-ri mine. Based on the results, soil texture may also affect soil homogeneity and friable asbestos exposure. Soils from the Jongmin-ri mine with sandy loam texture have more friable asbestos exposure than those from the Jecheon mine with loam to silt loam. It is well known that soil texture may be affected by water-holding capacity and soil moisture as well [24,25].

Water content in soils is also an important factor in the releasability of asbestos from soil to air. As shown in Figure 3, friable asbestos from soils increased with decreasing water contents. At the same wind velocity conditions, two to five times higher asbestos concentrations in air were found in soils with 10~20% water content rather than those with 20~30% water content. The same results were also reported by other studies [10,24,25].

### 3.2. Estimation of ELCR

Estimating risks from inhalation of asbestos would include the calculation of excess lifetime cancer risks (ELCR = EPC × TWF × IUR), where the exposure point concentration (EPC) means the concentration of asbestos fibers in the air (f/cc) for the specific activity being assessed, the time weighting factor (TWF) accounts for less-than-continuous exposure during a one-year exposure, and inhalation unit risk (IUR) is estimated by US EPA [24]. If the ELCR value does not exceed the threshold value (1×10^−4^), which is probably to be considered a safety zone.

As mentioned before, the modified RAS was designed to provide an alternative to activity-based sampling (ABS) for determining asbestos exposure in the air. In South Korea, the ABS method was used to measure the concentration of asbestos scattered through the soil disturbance activity in a specific scenario and determine the effect of asbestos scattering on the human body [24,29]. These asbestos exposure concentration scenarios included outdoor, indoor, and 11 ABS methods (bicycle, car, motorcycle, cultivator, walking, weeding, weed whacking, digging, field sweeping, physical training, and children playing in the dirt) suggested in the guidelines of the Korean government [24]. According to [29], the airborne naturally occurring asbestos (NOA) in agricultural activity (weeding, weed wracking, and digging), and daily activity (field sweeping, physical training, and children playing in the dirt) in the residents near abandoned asbestos mines in South Korea was 5.49 × 10^−2^ f/cc and 6.95 × 10^−2^ f/cc, respectively. The study also calculated the ELCR using the data and concluded that the ELCR of agricultural activity (9.71 × 10^−5^) was highest in the mid-east of South Korea, whereas that of daily life activity (1.08 × 10^−4^) was highest in the mid-west of South Korea. Thus, the ELCR exceeded the Korean criteria for soil remediation (0.0001) for the daily life activity of the mid-west of South Korea [29].

**Figure 4:**
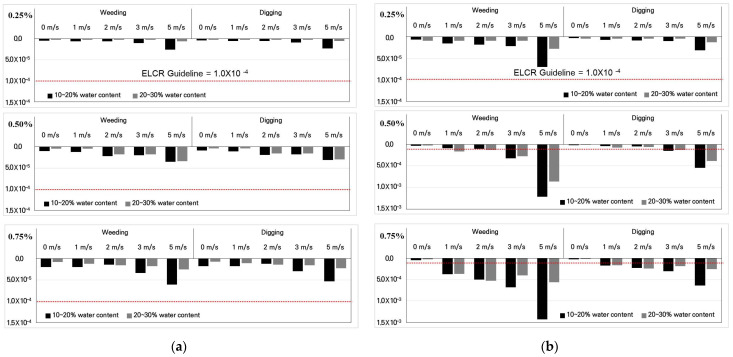
Calculated ELCR value as an asbestos concentration in soils from the Jecheon mine (**a**) and the Jongmin-ri mine (**b**).

In this study, only two scenarios were selected for farmland purposes, e.g., weeding to remove weeds and digging to transplant shrubs, for analyzing the environmental assessment of ELCR from asbestos exposure. To obtain the value of exposure time and exposure day, it conducted a question investigation on farmers who live nearby the mine area. Then, from the average data for how many times they work in the area, exposure time and exposure day could be estimated. Scenarios and the calculated ELCR values for the mines are shown in Table 2. In addition, the estimated ELCR values as asbestos contents in soils, scenarios, wind velocity, and water contents at the Jecheon mine and the Jongmin-ri mine are shown in Figure 4. Additional data for the calculated ELCR values are summarized in the Supplementary Materials of Tables S2 and S3 for the Jecheon mine and the Jongmin-ri mine, respectively.

As shown in the figures, the ELCR values decreased with increasing water contents from 10–20% to 20–30%, mainly due to differences in the releasability of asbestos from the soil surface. All of the estimated ELCR values from the Jecheon mine examined in various conditions were below the threshold value of 1 × 10^−4^. It can be evaluated that asbestos in soils from the mine may be safe in actions of weeding and digging for agricultural work. However, several estimated ELCR values from the Jongmin-ri mine examined various conditions were over the threshold value of 1 × 10^−4^. Especially, the ELCR values from soils with low contents of asbestos of 0.50% and high water content exceeded the threshold value due to high wind velocity. Therefore, it can be concluded that asbestos in soils from the mine may not be safe in the actions of weeding and digging for agricultural work around the Jongmin-ri mine. Consequently, proper remediation work for reducing friable asbestos in soils is needed in and around the mining area reported by [30].

## 4. Conclusions

This study has focused on the examination of the feasibility of the modified releasable asbestos sampler equipment, in the laboratory and field, controlled by wind velocity and water contents, and the findings can be concluded as follows:

(1) The modified releasable asbestos sampler (RAS) adopted in this study is easy and fast to collect asbestos releasability data under field conditions. In addition, the RAS can be easily controlled by water contents and wind velocity. Thus, the RAS can be used as an alternative method of ABS for evaluation on the risk assessment of asbestos in soils. The ABS is one of the more time-consuming and expensive processes for checking the environmental effects. In comparison with the ABS, the modified RAS is a more quick and cheap process for obtaining information on risk assessment;
(2) As a result of the releasable asbestos test by the modified RAS equipment from the Jecheon and Jongmin-ri mines, relatively high levels of friable asbestos were found in soils from the Jongmin-ri mine. This may be due to the high weathering process of the mine and soil texture. Soils from the Jongmin-ri mine were classified as sandy loam which has more friable asbestos exposure than those from the Jecheon mine with loam and silt loam. In addition, the releasability of asbestos from soil to the air increased with increasing wind velocity and decreasing water contents of soils;
(3) According to the estimation of ELCR by the two main scenarios for agricultural activity (weeding and digging) measured by the modified RAS, the ELCR values were below the threshold value of 1 × 10^−4^ in the Jecheon mine for 0.25–0.75% of asbestos-contained soils, which is probably to be considered a safety zone. In the Jongmin-ri mine, however, soils containing 0.50% and 0.75% of asbestos were over the threshold. This means that agricultural activity may promote the liberation and dispersion of fibres in the air, which may be correlated to excessive exposure. Therefore, it is strongly recommended that the Jongmin-ri mine area should be remediated as an adequate process for reducing releasable asbestos from soil to the air.

## Figures and Tables

**Table 1:** Asbestos concentrations, water contents, and soil texture of soils sampled from the Jecheon and Jongmin-ri mines.

Mine	Sample	Asbestos Concentration in Soil (%)		Water Contents (%)	Soil Texture (%)				Distance from the Mine (km)
					Sand	Silt	Clay	Texture	
Jecheonmine	JC01	0.25		18.9	41.8	45.6	12.6	loam	1.0
	JC02	0.50		14.9	36.9	53.9	9.2	silt loam	0.9
	JC03	0.75		16.5	42.8	51.8	5.4	silt loam	0.1
	All	Mean	0.43	18.9	43.8	45.8	10.4	loam and silt loam	0~1.5
	samples	Range	0.25~0.75	12.7~24.6	34.7~51.6	29.7~61.5	3.8~18.7		
	(n = 21)	STD	0.0020	2.9	4.9	6.9	3.8		
Jongmin-rimine	JM01	0.25		19.4	72.1	21.8	6.1	sandy loam	0.2
	JM02	0.50		17.5	65.7	20.7	13.6	sandy loam	0.1
	JM03	0.75		21.8	60.3	18.9	20.8	sandy loam	0.4
	All	Mean	0.42	21.4	60.1	26.6	13.2	sandy loam	0~1.0
	samples	Range	0.25~0.75	14.6~31.4	45.9~72.5	16.7~45.3	6.1~20.8		
	(n = 21)	STD	0.0017	4.5	8.2	7.7	4.3		

**Table 2:** Scenarios of the modified RAS and TWF and ELCR value for the Jecheon and Jongmin-ri mines.

Mine	Scenario	Time Weight Factor (TWF)			Inhalation Unit Risk (IUR)		
		Exposure Time (Hour/Day)	Exposure Frequency (Day/Year)	TWF	Initial Exposure (Year)	Exposure Period (Year)	IUR (f/cc)^−1^
Jecheon	Weeding	3.6	128.0	0.053	34.1	28.3	0.037
	Digging	1.8	138.3	0.028	25.5	31.8	0.060
Jongmin-ri	Weeding	2.8	150.6	0.048	26.0	36.6	0.063
	Digging	1.8	120.0	0.025	25.3	24.3	0.055

## Data Availability

The datasets used and/or analyzed during the current study are available from the corresponding author upon reasonable request.

## Supplementary Materials

The following supporting information can be downloaded at: https://www.mdpi.com/article/10.3390/toxics10120748/s1, Table S1: Data compilation of friable asbestos concentrations of tested soil in the two study areas, Table S2: Calculated ELCR value as asbestos concentration in soils from the Jecheon mine, Table S3: Calculated ELCR value as asbestos concentration in soils from the Jonmin-ri mine.
