# Supplementary material for: Environmental Assessment of Friable Asbestos from Soil to Air Using the Releasable Asbestos Sampler (RAS)
Source: Toxics. 2022 Dec 1;10(12):748. doi: 10.3390/toxics10120748 (PMC9782100; doi:10.3390/toxics10120748)
Supplement: Supplementary file 1 [file toxics-10-00748-s001.zip › toxics-1952852-supplementary.pdf]

Supplementary materials

Table S1. Data compilation of friable asbestos concentrations of tested soil in the two study areas.

Mine name	Asbestos in soils (%)	Wind velocity (m/s)	Friable asbestos in air (f/cc)	
			10~20% water content	20~30% water content
Jecheon	0.25	0	0.002	0.001
		1	0.003	0.001
		2	0.003	0.001
		3	0.005	0.001
		5	0.013	0.003
	0.50	0	0.005	0.002
		1	0.006	0.002
		2	0.011	0.009
		3	0.010	0.009
		5	0.018	0.017
	0.75	0	0.010	0.004
		1	0.010	0.006
		2	0.007	0.008
		3	0.017	0.009
		5	0.031	0.013
Jongmin-ri	0.25	0	0.002	0.003
		1	0.005	0.003
		2	0.006	0.003
		3	0.007	0.003
		5	0.023	0.009
	0.50	0	0.009	0.006
		1	0.027	0.053
		2	0.032	0.040
		3	0.105	0.089
		5	0.400	0.282
	0.75	0	0.013	0.007
		1	0.121	0.120
		2	0.163	0.173
		3	0.223	0.132
		5	0.471	0.185

Table S2. Calculated ELCR value as asbestos concentration in soils from the Jecheon mine.

Asbestos in soil(%)	Scenarios	Wind velocity (m/s)	Calculated ELCR	
			10~20% water content	20~30% water content
0.25	Weeding	0	3.89E-06	1.95E-06
		1	5.84E-06	1.95E-06
		2	5.84E-06	1.95E-06
		3	9.73E-06	1.95E-06
		5	2.53E-05	5.84E-06
	Digging	0	3.41E-06	1.71E-06
		1	5.12E-06	1.71E-06
		2	5.12E-06	1.71E-06
		3	8.53E-06	1.71E-06
		5	2.22E-05	5.12E-06
0.50	Weeding	0	9.73E-06	3.89E-06
		1	1.17E-05	3.89E-06
		2	2.14E-05	1.75E-05
		3	1.95E-05	1.75E-05
		5	3.50E-05	3.31E-05
	Digging	0	8.53E-06	3.41E-06
		1	1.02E-05	3.41E-06
		2	1.88E-05	1.53E-05
		3	1.71E-05	1.53E-05
		5	3.07E-05	2.90E-05
0.75	Weeding	0	1.95E-05	7.79E-06
		1	1.95E-05	1.17E-05
		2	1.36E-05	1.56E-05
		3	3.31E-05	1.75E-05
		5	6.03E-05	2.53E-05
	Digging	0	1.71E-05	6.82E-06
		1	1.71E-05	1.02E-05
		2	1.19E-05	1.36E-05
		3	2.90E-05	1.53E-05
		5	5.29E-05	2.22E-05

Table S3. Calculated ELCR value as asbestos concentration in soils from the Jonmin-ri mine.

Asbestos in soil(%)	Scenarios	Wind velocity (m/s)	Calculated ELCR	
			10~20% water content	20~30% water content
0.25	Weeding	0	6.07E-06	9.10E-06
		1	1.52E-05	9.10E-06
		2	1.82E-05	9.10E-06
		3	2.12E-05	9.10E-06
		5	6.98E-05	2.73E-05
	Digging	0	2.71E-06	4.07E-06
		1	6.78E-06	4.07E-06
		2	8.14E-06	4.07E-06
		3	9.49E-06	4.07E-06
		5	3.12E-05	1.22E-05
0.50	Weeding	0	2.73E-05	1.82E-05
		1	8.19E-05	**1.61E-04**
		2	9.70E-05	**1.21E-04**
		3	**3.18E-04**	**2.70E-04**
		5	**1.21E-03**	**8.55E-04**
	Digging	0	1.22E-05	8.14E-06
		1	3.66E-05	7.19E-05
		2	4.34E-05	5.42E-05
		3	**1.42E-04**	**1.21E-04**
		5	**5.42E-04**	**3.82E-04**
0.75	Weeding	0	3.94E-05	2.12E-05
		1	**3.67E-04**	**3.64E-04**
		2	**4.94E-04**	**5.25E-04**
		3	**6.76E-04**	**4.00E-04**
		5	**1.43E-03**	**5.61E-04**
	Digging	0	1.76E-05	9.49E-06
		1	**1.64E-04**	**1.63E-04**
		2	**2.21E-04**	**2.35E-04**
		3	**3.02E-04**	**1.79E-04**
		5	**6.39E-04**	**2.51E-04**

Letters in bold and underline are over the guideline (1.00E-04) of ELCR.
